# Does *de novo* malignancy heighten the risk of rejection in kidney transplant recipients?

**DOI:** 10.1093/ckj/sfae349

**Published:** 2024-11-19

**Authors:** Erol Demir, Mevlut Tamer Dincer, Cebrail Karaca, Cansu Erel, Latif Karahan, Aslihan Pekmezci, Sinan Trabulus, Nurhan Seyahi, Aydin Turkmen

**Affiliations:** Transplant Immunology Research Center of Excellence, Koç University Hospital, Koç University, Istanbul, Türkiye; Division of Nephrology, Department of Internal Medicine, Cerrahpaşa Faculty of Medicine, Istanbul University Cerrahpaşa, Istanbul, Türkiye; Division of Nephrology, Department of Internal Medicine, Cerrahpaşa Faculty of Medicine, Istanbul University Cerrahpaşa, Istanbul, Türkiye; Division of Nephrology, Department of Internal Medicine, Istanbul Faculty of Medicine, Istanbul University, Istanbul, Türkiye; Division of Nephrology, Department of Internal Medicine, Istanbul Faculty of Medicine, Istanbul University, Istanbul, Türkiye; Division of Nephrology, Department of Internal Medicine, Cerrahpaşa Faculty of Medicine, Istanbul University Cerrahpaşa, Istanbul, Türkiye; Division of Nephrology, Department of Internal Medicine, Cerrahpaşa Faculty of Medicine, Istanbul University Cerrahpaşa, Istanbul, Türkiye; Division of Nephrology, Department of Internal Medicine, Cerrahpaşa Faculty of Medicine, Istanbul University Cerrahpaşa, Istanbul, Türkiye; Division of Nephrology, Department of Internal Medicine, Istanbul Faculty of Medicine, Istanbul University, Istanbul, Türkiye

**Keywords:** allograft rejection, chemotherapy, kidney transplantation, malignancies, survival analysis

## Abstract

**Background:**

Malignancies are the third leading cause of death among kidney transplant recipients. These patients face increased mortality and challenges such as allograft loss and rejection, which may arise from surgical complications, changes in immunosuppressive therapy or the use of chemotherapeutics. This study aims to examine the risk of allograft rejection and loss in kidney transplant recipients diagnosed with *de novo* malignancies.

**Methods:**

This retrospective case–control study included adult kidney transplant patients from 1986 to 2020 who developed *de novo* malignancies. Each patient with a malignancy was matched with a control without malignancy using the nearest neighbor matching method. The outcomes measured were biopsy-confirmed allograft rejection, death-censored allograft loss and overall mortality after the diagnosis of malignancy in the malignancy group and at any point in the control group.

**Results:**

Of 2750 records reviewed, 267 patients (9.7%) had biopsy-confirmed malignancies, with a median age of 60 years and 66.3% men. The median follow-up was 218 months. Kaplan–Meier analysis showed that the allograft rejection rates were lower in the malignancy group compared with the control group (26 vs 60, *P *< .001). Overall mortality was higher in the malignancy group, although this difference was not statistically significant (104 vs 73, *P *= .25). Death-censored allograft loss was similar between groups (22 vs 32, *P *= .49). Chemotherapy and older recipient age were associated with reduced allograft rejection risk, as indicated by multivariable regression analysis.

**Conclusions:**

In kidney transplant recipients with *de novo* malignancies, death with a functioning graft remains significant. However, allograft loss rates do not increase compared with those without malignancies, and rejection risk is reduced, especially in older and chemotherapy-treated patients. These findings suggest that managing immunosuppression reduction in this population may be appropriate, but further research is needed to determine optimal care strategies.

## Graphical Abstract

**Graphical Abstract:**
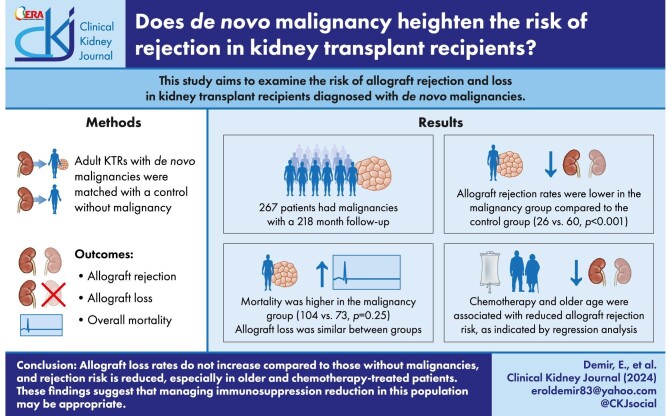

KEY LEARNING POINTS **What was known:**

- Kidney transplant recipients face a higher risk of new malignancies compared with dialysis patients, significantly impacting mortality and allograft survival.
- Previous studies indicated an increased risk of allograft rejection with the use of immune checkpoint inhibitors.
- The extent of allograft rejection and loss risk following malignancy diagnosis in kidney transplant recipients remained unclear.

**This study adds:**

- Kidney transplant recipients with *de novo* malignancies have a lower rate of allograft rejection compared with non-malignant recipients.
- Chemotherapy and older age are associated with a reduced risk of allograft rejection in these patients.
- Immunosuppression management can be tailored for older patients and those undergoing chemotherapy to optimize care.

**Potential impact:**

- This study suggests that immunosuppression reduction in older and chemotherapy-treated kidney transplant recipients with malignancies may be safely managed.
- These findings can guide clinical decisions and policies to enhance the care and outcomes for kidney transplant recipients diagnosed with malignancies.
- Future research could focus on optimizing immunosuppressive therapy and monitoring protocols for these vulnerable patients.

## INTRODUCTION

Kidney transplantation is the preferred treatment for individuals with end-stage renal disease, offering significant improvements in patient survival and quality of life [1]. However, kidney transplant recipients face a 2- to 3-fold higher risk of developing new malignancies compared with dialysis patients [2]. Malignancies are the third leading cause of death in these patients, following infections and cardiovascular diseases [3].

The development of malignancies in kidney transplant recipients presents unique challenges. These patients not only face increased mortality with a functioning graft but also encounter challenges, such as allograft loss and rejection. Allograft loss can arise from post-surgical complications related to kidney tumors or rejection induced by changes in immunosuppressive therapy or chemotherapeutics, including immune checkpoint inhibitors [4, 5].

Despite these challenges, it remains unclear to what extent the risk of allograft rejection and loss is increased in kidney transplant recipients after malignancy diagnosis compared with non-malignant kidney transplant recipients with similar follow-up periods. This study aims to examine the incidence of allograft loss and rejection in kidney transplant recipients following the diagnosis of *de novo* malignancies.

## MATERIALS AND METHODS

### Study design and population

This retrospective case–control study was conducted across two kidney transplantation centers at the university hospital. The inclusion criteria encompassed adult patients (above 18 years of age) diagnosed with malignancy who underwent kidney transplantation between 1986 and 2020. Of the 2750 eligible patient files, 11 individuals with a history of pre-transplant malignancy were excluded. Initially, 278 patients were diagnosed with malignancies, after applying the exclusion criteria, 267 patients were included in the analysis. Each patient with malignancy was matched 1:1 with a kidney transplant recipient without malignancy using the nearest neighbor matching method, considering the post-transplant follow-up period (Supplementary data, Fig. S1).

### Statement of ethics

The study protocol was approved by the Clinical Research Ethics Committee of Istanbul University Cerrahpaşa with the number 188 137 on 09.12.2019 and adhered to the 1975 Declaration of Helsinki, revised in 2013. Due to the purely observational nature of the study, the need for informed consent was waived by an independent institutional review board.

### Data collection

Data on demographic and clinical parameters were extracted from patient records and electronic databases. These included information on age, gender, smoking and alcohol use, the etiology of end-stage renal disease, history of diabetes mellitus, type, duration of pre-transplant renal replacement therapy, transplantation date, donor age, donor type, number of human leukocyte antigen (HLA) mismatches, induction treatment, maintenance immunosuppressive therapy, rejection episodes, family history of malignancy, type of malignancy, the interval between transplantation and malignancy, duration of rejection following transplantation, treatment of rejection episodes, treatment regimen for malignancy, and dates of death or the last follow-up.

### Immunosuppressive protocols

Each center followed the same immunosuppressive regimen protocol. A desensitization protocol was employed if a positive B or T cell flow cytometric crossmatch was detected. Every patient received induction therapy comprising either rabbit anti-thymocyte globulin or basiliximab, selected based on the patient's immunological risk profile. The risk assessment considered factors such as the presence and quantity of donor-specific antibodies (DSA), number of HLA mismatches, donor type, and history of blood transfusions, pregnancy and prior transplants. Maintenance immunosuppressive therapy consisted of a combination of a calcineurin inhibitor (tacrolimus or cyclosporine), an antimetabolite (mycophenolate mofetil, mycophenolate sodium or azathioprine), mammalian target of rapamycin (mTOR) (everolimus or sirolimus) and prednisolone.

### Malignancy screening protocols

The same malignancy screening protocols were applied before and after transplantation at both centers. Before transplantation, males underwent prostate-specific antigen testing and digital prostate examinations, while females received breast and pelvic examinations, mammography and PAP smears. Individuals over 50 years old underwent fecal occult blood tests, abdominal ultrasounds and chest X-rays, and current or former smokers underwent low-dose thorax computed tomography. After transplantation, patients underwent dermatological examinations at every clinic visit. Additionally, males received annual prostate-specific antigen testing and digital prostate examinations, while females underwent breast and pelvic examinations, mammography and PAP smears. Individuals over 50 years old received annual fecal occult blood tests and colonoscopies every 5 years, along with annual abdominal ultrasounds and chest X-rays.

### In the presence of malignancy

Following a malignancy diagnosis, maintenance immunosuppression was adjusted based on the tumor type and stage, and the patient's immunological risk profile. For non-melanoma skin cancers, such as squamous cell carcinoma, calcineurin inhibitors or antimetabolites were typically reduced or replaced with mTOR inhibitors. In contrast, no changes were generally made for patients with low-grade skin tumors, like basal cell carcinoma. For solid tumors, calcineurin inhibitors therapy or antimetabolites were reduced in patients undergoing surgical treatment for early-stage tumors. However, for advanced-stage tumors requiring chemotherapy, calcineurin inhibitors or antimetabolites were discontinued. In cases of post-transplant lymphoproliferative disease, calcineurin inhibitors and antimetabolites were omitted during rituximab-based chemotherapy, with steroid therapy serving as the sole form of immunosuppression.

Regarding the diagnosis of rejection, allograft biopsies were performed in the presence of new-onset proteinuria or allograft dysfunction and were assessed by pathologists at each center, following the Banff classifications.

For this study, malignancies were categorized into three groups: skin cancer, which includes both melanoma and non-melanoma types; solid organ malignancies; and post-transplant lymphoproliferative disorder.

### Outcomes

The primer outcome was on biopsy-proven allograft rejection, observed after the diagnosis of malignancy in the malignancy group and at any point in the control group. Secondary outcomes encompassed overall mortality and death-censored allograft loss, occurring after the diagnosis of malignancy in the malignancy group and at any time in the control group.

### Statistical analysis

Categorical data, including recipient and donor gender, donor type, etiology of end-stage renal disease, history of smoking and alcohol use, presence of diabetes mellitus, type of pretransplant renal replacement therapy, HLA mismatch, induction, and maintenance immunosuppressive therapy, treatment regimen for rejection episodes and treatment protocol for malignancy, were analyzed using the chi-square or Fisher's exact tests. The results were presented in terms of counts and proportions. Continuous variables, such as duration of the post-transplant follow-up period, the time of previous renal replacement therapies, the interval between transplantation and malignancy, duration of rejection following transplantation, and ages of both recipients and donors were summarized using medians and interquartile ranges. The Shapiro–Wilk test was employed to assess the normality of the data distribution. For quantitative variables with nonparametric distributions, the Mann–Whitney U test was applied.

Univariate logistic regression models were utilized to identify potential risk factors for allograft rejection among patients with malignancy. Various potential confounding factors, including the use of erythropoietin-stimulating agents before transplantation, re-transplantation, receiving a kidney from a living donor, induction with anti-thymocyte globulin, HLA mismatch, donor age, recipient age, desensitization, use of chemotherapy in malignancy treatment, conversion from calcineurin inhibitors or antimetabolite to mTOR, as well as the presence of cytomegalovirus (CMV) and BK virus infections, were entered into this model. A multivariate risk model was constructed using significant confounders identified through the univariate analysis. Odds ratios (ORs) and their corresponding 95% confidence intervals (CIs) were calculated to represent the change in risk per unit increase in a continuous variable.

The Kaplan–Meier curve was also employed to estimate allograft rejection, overall mortality rates and death-censored allograft loss for patients and the control group. The survival analysis for patients and death-censored allograft loss between the two groups at the 5th, 10th and 20th years utilized the log-rank test. A significance level of <.05 was considered statistically significant.

## RESULTS

### Baseline characteristics at the time of transplantation

A total of 267 kidney transplant recipients (9.7%) had biopsy-confirmed *de novo* malignancies after transplantation. The median age of these patients at the time of transplantation was 60 years [interquartile range (IQR) 22–83], with 177 (66.3%) being male (Table 1). The patients were monitored for a median duration of 218 months (IQR 14–547) following transplantation for all outcomes. The most common etiologies of chronic kidney disease were chronic glomerulonephritis (21.0%), hypertension (7.9%) and congenital anomalies of the kidney and urinary tract (CAKUT) (7.9%), followed by polycystic kidney disease (7.5%) and diabetes mellitus (4.1%). Donors were living relatives in 186 cases (69.7%).

**Table 1:** Demographic and transplantation characteristics of malignancy and control groups.

	Malignancy group (*n* = 267)	Control group (*n* = 267)	*P*-value
Age at the time of transplantation (years), median (IQR)	60 (22–83)	55 (25–83)	**<.001**
Gender, male (*n*, %)	177 (66.3)	183 (68.5)	.58
Post-transplant follow-up (months), median (IQR)	218 (14–547)	200 (1–514)	.15
Etiology of CKD (*n*, %)			
Chronic glomerulonephritis	56 (21.0)	73 (27.3)	.08
Hypertension	21 (7.9)	15 (5.6)	
CAKUT	21 (7.9)	19 (7.1)	
Polycystic kidney disease	20 (7.5)	10 (3.8)	
Diabetes mellitus	11 (4.1)	4 (1.5)	
Other	44 (16.4)	39 (14.6)	
Unknown	94 (35.2)	107 (40.1)	
Malignancy in the family (*n*, %)	34 (12.7)	3 (1.1)	**<.001**
Habits (*n*, %)			
Smoking	70 (26.2)	46 (17.2)	**.011**
Alcohol	15 (5.7)	7 (2.6)	.08
Type of donor, living (*n*, %)	186 (69.7)	194 (72.6)	.26
Donor age (years), median (IQR)	45 (14–84)	42 (9–77)	.18
Type of previous RRT (*n*, %)			
Hemodialysis	194 (72.7)	200 (74.9)	.71
Peritoneal dialysis	18 (6.7)	22 (8.2)	
HD + PD	13 (4.9)	13 (4.9)	
Transplantation	5 (1.9)	5 (1.9)	
Duration of previous RRT (months), median (IQR)	20 (1–324)	19 (1–312)	.53

Bold values denote statistical significance at the *P* < .05 level.

CAKUT, congenital anomalies of the kidneys and urinary tracts; CKD, chronic kidney disease; HD; hemodialysis; PD, peritoneal dialysis; RRT, renal replacement therapy.

### Types of malignancy in the malignancy group

In the malignancy group, the types of malignancies were distributed as follows: solid organ malignancies accounted for 45.3%, skin cancer for 42.3% and post-transplant lymphoproliferative disorder for 12.4% (Table 2). Non-melanoma skin cancer was prevalent, representing 40.1% of cases. Notably, the most common solid organ tumors were urinary tract cancer (17.6%), gastrointestinal tract cancer (6.7%) and breast cancer (6.0%). Kaposi's sarcoma had a prevalence of 10.1%. The median time from kidney transplantation to malignancy diagnosis was 127 months (IQR 13–507). For the treatment of malignancy, 125 patients (46.8%) received only surgical treatment, 50 patients (18.7%) received a combination of surgery, chemotherapy and radiotherapy, 27 patients (10.1%) received only chemotherapy, 5 patients (1.9%) received a combination of chemotherapy and radiotherapy, and only 3 patients (1.1%) received only radiotherapy. None of the patients received immune checkpoint inhibitors.

**Table 2:** Parameters about malignancy in the malignancy group.

	*n* = 267	%
Type of malignancy (*n*, %)		
Solid tumors	121	45.3
Urogenital system	47	17.6
Gastrointestinal tract	18	6.7
Breast	16	6.0
Thyroid gland	13	4.9
Lung	7	2.6
CNS	7	2.6
Other	10	3.7
Unknown primary	3	1.1
Skin cancer	113	42.3
Non-melanoma	107	40.1
Melanoma	6	2.2
PTLD	33	12.4
The interval between Tx and malignancy (months), median (IQR)	127 (13–507)	
Treatment regimen for malignancy		
Surgery only	125	46.8
Surgery chemotherapy and radiotherapy	50	18.7
Chemotherapy	27	10.1
Chemotherapy and radiotherapy	5	1.9
Radiotherapy	3	1.1
Change of immunosuppressive regimen (*n*, %)		
Conversion from CNI or antimetabolite to mTOR	110	41.2
Discontinuation of CNI or antimetabolite	29	10.9
Withdrawal of CNI and antimetabolite	12	4.5
No change	82	30.7

CNI, calcineurin inhibitor; CNS, central nervous system; PTLD, posttransplant lymphoproliferative disorder; Tx, transplantation.

### Outcomes

The median time from malignancy to rejection was 72 months (IQR 2–217). The incidence of allograft rejection rates was significantly higher in the control group than in the malignancy group [26 (9.7%) patients in the malignancy group and 60 (22.5%) in the control group, *P *< .001]. The median time from transplant to rejection was longer in the malignancy group than in the control group (142 months in the malignancy group vs 60 months in the control group, *P *= .027). Also, the malignancy group had a higher mortality rate compared with the control group [104 (40.1%) vs 73 (27.3%), *P *= .004]. Although the most common cause of death remained unknown in both groups, the incidence of malignancy-related multiple organ failure was significantly higher in the malignancy group compared with the control group. Conversely, the control group exhibited a significantly higher incidence of respiratory failure due to chronic obstructive pulmonary disease (COPD) compared with the malignancy group (both comparisons yielded *P* < .001). In addition, death-censored allograft loss was similar between the groups [22 (8.2%) patients in the malignancy group and 32 (12.0%) in the control group, *P *= .15]. These outcomes are summarized in Table 3.

**Table 3:** Study parameters and outcomes in the malignancy and control group.

	Malignancy group (*n* = 267)	Control group (*n* = 267)	*P*-value
Type of induction therapy (*n*, %)			
ATG	65 (24.3)	49 (18.4)	.083
Basiliximab	19 (7.1)	16 (6.0)	
None	107 (40.1)	136 (50.9)	
Unknown	76 (28.5)	66 (24.7)	
HLA mismatch, median (IQR)	3 (0–6)	3 (0–6)	.50
Usage of ESA (*n*, %)			
Before Tx	65 (24.3)	32 (11.9)	**<.001**
After Tx	45 (16.9)	53 (19.8)	.37
Post-Tx diabetes mellitus (*n*, %)	7 (2.6)	65 (24.3)	**<.001**
Initial maintenance IS regimen (*n*, %)			
Steroids	255 (95.5)	258 (97.3)	.50
Tacrolimus	88 (33.0)	78 (29.2)	.34
Cyclosporine A	107 (40.1)	163 (61.0)	**<.001**
Mycophenolate derivatives	155 (58.1)	140 (52.4)	.19
Azathioprine	80 (30.0)	111 (41.6)	**.005**
mTOR inhibitors	20 (7.5)	9 (3.3)	**.035**
Allograft rejection (*n*, %)	26 (9.7)	60 (22.5)	<.001
T-cell mediated	14 (5.2)	40 (15.0)	
Antibody mediated	12 (4.5)	20 (7.5)	
The median time from Tx to rejection (months), median (IQR)	142 (14–273)	60 (11–240)	**.027**
Treatment of rejection (*n*, %)			
Steroids	9 (3.4)	16 (6.0)	.14
ATG + steroid	1 (0.4)	3 (1.1)	
IVIG + PP	12 (4.5)	8 (3.0)	
IVIG + PP + ATG + steroid	2 (0.7)	0	
Death-censored graft loss (*n*, %)	22 (8.2)	32 (12.0)	.15
Death with functioning graft (*n*, %)	100 (37.5)	61 (22.8)	**<.001**
Overall mortality (*n*, %)	104 (40.1)	73 (27.3)	**.004**
Cause of death (*n*, %)			
Respiratory failure	4 (3.8)	25 (34.2)	**<.001**
MSOF	25 (24.0)	0	**<.001**
Infections	19 (18.2)	6 (8.2)	.18
Ischemic heart disease	8 (7.7)	2 (2.7)	.20
Cerebrovascular disease	2 (1.9)	1 (1.4)	1
Unknown	46 (44.2)	39 (53.4)	.23

Bold values denote statistical significance at the *P* < .05 level.

ATG, anti-thymocyte globulin; ESA, erythropoietin-stimulating agents; IS, immunosuppression; IVIG, intravenous immunoglobulin; MSOF, malignancy-related multi-organ disorder; PP, plasmapheresis; RTX, rituximab; Tx, transplantation.

### Survival rates

The survival rates for the malignancy group were similar to those of the control group at the 5th (94% vs 94%; *P *= .70), 10th (78% vs 82%; *P *= .349) and 20th year (60% vs 76%; *P *= .570), and these differences were not statistically significant. Regarding death-censored allograft survival rates, there were no differences in the 5th year (99% vs 99%; *P *= .866), the 10th year (98% vs 98%; *P *= .834) and the 20th year (78% vs 62%; *P *= .355) between the groups.

The Kaplan–Meier curve analysis showed that allograft rejection rates were higher in the control group compared with the malignancy group (*P *< .001) (Supplementary data, Fig. S2). Although overall mortality rates were higher in the malignancy group compared with the control group, this difference did not reach statistical significance (*P *= .25) (Supplementary data, Fig. S3). In addition, the study found that death-censored allograft loss was similar between the groups (*P *= .49) (Supplementary data, Fig. S4).

### Exploration of risk factors

Univariate regression analysis did not establish significant relationships between allograft rejection and various factors, including living donor, HLA mismatch, donor age, desensitization, and CMV/BKV infection before rejection. Nevertheless, using chemotherapy [OR 0.269 (95% CI 0.093–0.780)] during malignancy treatment and older recipient age [OR 0.971 (95% CI 0.949–0.994)] was associated with a noteworthy reduction in the risk of allograft rejection, as indicated by multivariable regression analysis (Table 4).

**Table 4:** Regression analysis investigating confounders associated with allograft rejection.

	Univariate analysis			Multivariate analysis		
	OR	CI	*P*-value	OR	CI	*P*-value
Use of ESA before Tx	1.21	0.55–2.66	.64			
Re-transplantation	**4.600**	**1.720–12.290**	**.002**	2.99	0.90–9.98	.08
Living donor	1.00	0.61–1.66	.99			
ATG induction	**2.710**	**1.230–5.970**	**.013**	0.56	0.27–1.14	.11
HLA mismatch	0.98	0.75–1.27	.88			
Donor age	0.98	0.96–1.01	.17			
Recipient age	**0.980**	**0.960–1.010**	**.056**	**0.971**	**0.949–0.989**	**.015**
Desensitization	3.60	0.45–29.00	.23			
Using chemo in the malignancy treatment	**0.230**	**0.080–0.650**	**.005**	**0.269**	**0.093–0.780**	**.016**
Change in immunosuppression after malignancy diagnosis	0.42	0.18–1.006	.052			
CMV infection	1.55	0.81–2.95	.18			
BKV infection	1.04	0.12–9.03	.97			

Bold values denote statistical significance at the *P* < .05 level.

ATG, antithymocyte globulin; chemo, chemotherapy; CNI, calcineurin inhibitor; ESA, erythropoietin-stimulating agents; IS, immunosuppression; Tx, transplantation.

## DISCUSSION

In this study, we delved into the occurrence of allograft rejection in kidney transplant recipients with malignancies, exploring an area of research that had not previously been investigated before the era of checkpoint inhibitors. We identified biopsy-confirmed malignancies in 267 kidney transplant recipients, with a median age of 60 years, comprising 177 men. These patients had a median post-transplant follow-up period of 218 months. Based on the Kaplan–Meier analysis, the allograft rejection rates were lower in the malignancy group compared with the control group. However, the observed disparity in overall mortality between the two groups did not achieve statistical significance. In addition, individuals with malignancies displayed similar death-censored allograft loss in contrast to those without malignancies. Furthermore, our multivariable regression analysis demonstrated that the use of chemotherapy during malignancy treatment and older age was associated with a noteworthy reduction in the risk of allograft rejection.

Previous research has indicated an increased risk of allograft rejection in kidney transplant recipients with malignancies, particularly when treated with immune checkpoint inhibitors [6]. However, the phase 1 trial of nivolumab, an immune checkpoint inhibitor, reported allograft rejection in only two cases (11.8%), with graft loss in one patient (5.9%) [7]. On the other hand, our study revealed a lower allograft rejection rate in kidney transplant recipients with malignancies group compared with the control group. Several factors may account for these findings. First, anti-tumor therapies such as chemotherapy may have offered protection against allograft rejection by influencing the count and function of B and T cells. Second, the difference could be linked to impaired effector and regulator lymphocyte function in kidney transplant recipients with malignancies [8]. Third, the higher median age of the malignancy group compared with the control group may explain the lower incidence of rejection observed in older patients. Furthermore, it is important to note that the patients involved in this study were not administered any immune checkpoint inhibitors. Consequently, conflicting findings regarding rejection frequency in kidney transplant recipients with malignancy have emerged, underscoring the necessity for additional prospective studies to provide more comprehensive insights into this subject.

Traditionally, risk factors for allograft rejection include HLA mismatch, older donors, absence of induction therapy and desensitization [9]. However, our study did not observe many of these factors significantly impacting rejection rates. This deviation may be attributed to impaired lymphocyte function, the relatively low number of allograft rejections and the high mortality rates. Moreover, our patient population, in contrast to the Western cohort, comprises individuals with lower immunological risk, more living donor transplants, fewer HLA mismatches and shorter periods of dialysis. Nevertheless, this result should be validated in further studies due to the relatively small number of patients and low rejection rates.

Malignancy poses a substantial risk of mortality in kidney transplant recipients. In our study, the 10-year survival rate for kidney transplant recipients with malignancies was 78%, within the 73%–88% range reported in the literature [10–18]. Discrepancies in these figures can be attributed to differences in the duration of pre-transplant dialysis, the frequency of fatal cancer cases, and the proportion of living donors, all of which varied between our study and existing literature. As anticipated, the primary causes of death in the malignancy group were related to multiple organ failure, infections and ischemic heart disease, influenced by both the cancer and its treatment. In contrast, the control group exhibited a significantly higher incidence of respiratory failure, likely due to smoking-related COPD, as these patients did not suffer from malignancy-related complications.

Malignancies are known to progress more rapidly and carry a higher mortality rate in kidney transplant recipients compared with the general population [19]. In our study, surgical interventions were primarily utilized for non-melanoma skin cancers and, to a lesser extent, for early-stage solid tumors. However, nearly half of the patients with advanced-stage malignancies were identified, necessitating multimodal treatments, including surgery, chemotherapy and radiotherapy. This likely reflects the tendency for malignancies in this population to be diagnosed at more advanced stages, requiring complex therapeutic approaches. The late-stage diagnosis and increased treatment demands in kidney transplant recipients may be linked to impaired function of B and T lymphocytes, as well as natural killer cells, which play a critical role in tumor apoptosis [20]. Furthermore, the observed reduction in rejection rates among these patients suggests a weakened immune response, which may contribute to poorer tumor outcomes.

While our study provides valuable insights, it has several limitations. Selection biases may have influenced treatment modalities in the malignancy group due to its retrospective and non-randomized nature. Additionally, the absence of monitoring for anti-HLA antibodies during the study period makes it impossible to gauge their role in these patients. Subclinical rejections might also have been underdiagnosed due to the absence of protocol biopsies. The lack of a power analysis raises the possibility of type II statistical error. On the positive side, the study's strength lies in its median follow-up period of over 15 years.

In conclusion, for kidney transplant recipients with *de novo* malignancies, death with a functioning graft remains a significant issue. However, the rate of allograft loss in this population does not increase compared with similar patients without malignancies. Notably, the risk of rejection is reduced, particularly in older patients and those undergoing chemotherapy. These findings suggest that immunosuppression reduction might be managed in older and chemotherapy-treated patients, providing a basis for further research in optimizing care for these vulnerable patients.

## Supplementary Material

sfae349_Supplemental_File

## Data Availability

The data of this study are available from the corresponding author upon reasonable request.

## References

[1] McDonald SP, Russ GR. Survival of recipients of cadaveric kidney transplants compared with those receiving dialysis treatment in Australia and New Zealand, 1991–2001. Nephrol Dial Transplant 2002;17:2212–9. 10.1093/ndt/17.12.2212 12454235

[2] Stewart JH, Vajdic CM, van Leeuwen MT et al. The pattern of excess cancer in dialysis and transplantation. Nephrol Dial Transplant 2009;24:3225–31. 10.1093/ndt/gfp331 19589786

[3] Awan AA, Niu J, Pan JS et al. Trends in the causes of death among kidney transplant recipients in the United States (1996-2014). Am J Nephrol 2018;48:472–81. 10.1159/000495081 30472701 PMC6347016

[4] Serkies K, Dębska-Ślizień A, Kowalczyk A et al. Malignancies in adult kidney transplant candidates and recipients: current status. Nephrol Dial Transplant 2023;38:1591–602. 10.1093/ndt/gfac239 35998321

[5] Hellemans R, Pengel LHM, Choquet S et al.; for ESOT Workstream 3 of the TLJ (Transplant Learning Journey) project. Managing immunosuppressive therapy in potentially cured post-kidney transplant cancer (excluding non-melanoma skin cancer): an overview of the available evidence and guidance for shared decision-making. Transpl Int 2021;34:1789–800. 10.1111/tri.13952 34146426 PMC8518116

[6] Nguyen LS, Ortuno S, Lebrun-Vignes B et al. Transplant rejections associated with immune checkpoint inhibitors: a pharmacovigilance study and systematic literature review. Eur J Cancer 2021;148:36–47. 10.1016/j.ejca.2021.01.038 33721705

[7] Carroll RP, Boyer M, Gebski V et al. Immune checkpoint inhibitors in kidney transplant recipients: a multicentre, single-arm, phase 1 study. Lancet Oncol 2022;23:1078–86. 10.1016/S1470-2045(22)00368-0 35809595

[8] Hope CM, Coates PT, Carroll RP. Immune profiling and cancer post transplantation. World J Nephrol 2015;4:41–56. 10.5527/wjn.v4.i1.41 25664246 PMC4317627

[9] Pratschke J, Dragun D, Hauser IA et al. Immunological risk assessment: the key to individualized immunosuppression after kidney transplantation. Transplant Rev (Orlando) 2016;30:77–84. 10.1016/j.trre.2016.02.002 26965071

[10] Rousseau B, Guillemin A, Duvoux C et al. Optimal oncologic management and mTOR inhibitor introduction are safe and improve survival in kidney and liver allograft recipients with de novo carcinoma. Int J Cancer 2019;144:886–96. 10.1002/ijc.31769 30155929

[11] Karatas M, Okut G, Simsek C et al. Genitourinary cancers following kidney transplant: our 20 years of experience with mechanistic target of rapamycin inhibitors. Exp Clin Transplant 2022;20:145–8. 10.6002/ect.MESOT2021.P72 35384826

[12] Cheung CY, Man Ma MK, Chak WL et al. Conversion to mammalian target of rapamycin inhibitors in kidney transplant recipients with de novo cancers. Oncotarget 2017;8:44833–41. 10.18632/oncotarget.14908 28160552 PMC5546523

[13] Chiurchiu C, Carreño CA, Schiavelli R et al.; Argentinian Registry of Everolimus Treated Renal Transplant Recipients. Results of the conversion to everolimus in renal transplant recipients with posttransplantation malignancies. Transplant Proc 2010;42:277–9. 10.1016/j.transproceed.2009.11.017 20172329

[14] Budde K, Lehner F, Sommerer C et al.; ZEUS Study Investigators. Five-year outcomes in kidney transplant patients converted from cyclosporine to everolimus: the randomized ZEUS study. Am J Transplant 2015;15:119–28. 10.1111/ajt.12952 25521535

[15] Au EH, Chapman JR, Craig JC et al. Overall and site-specific cancer mortality in patients on dialysis and after kidney transplant. J Am Soc Nephrol 2019;30:471–80. 10.1681/ASN.2018090906 30765426 PMC6405152

[16] Imamura R, Nakazawa S, Yamanaka K et al. Cumulative cancer incidence and mortality after kidney transplantation in Japan: a long-term multicenter cohort study. Cancer Med 2021;10:2205–15. 10.1002/cam4.3636 33314709 PMC7982608

[17] Teo SH, Lee KG, Lim GH et al. Incidence, risk factors and outcomes of malignancies after kidney transplantation in Singapore: a 12-year experience. Singapore Med J 2019;60:253–9. 10.11622/smedj.2018122 30311626 PMC6535454

[18] Fröhlich FA, Halleck F, Lehner L et al. De-novo malignancies after kidney transplantation: a long-term observational study. PLoS One 2020;15:e0242805. 10.1371/journal.pone.0242805 33253202 PMC7703884

[19] Ietto G, Gritti M, Pettinato G et al. Tumors after kidney transplantation: a population study. World J Surg Oncol 2023;21:18. 10.1186/s12957-023-02892-3 36691019 PMC9869548

[20] Peraldi MN, Berrou J, Venot M et al. Natural killer lymphocytes are dysfunctional in kidney transplant recipients on diagnosis of cancer. Transplantation 2015;99:2422–30. 10.1097/TP.0000000000000792 26798861

