# Supplementary material for: Does de novo malignancy heighten the risk of rejection in kidney transplant recipients?
Source: Clin Kidney J. 2024 Nov 19;17(12):sfae349. doi: 10.1093/ckj/sfae349 (PMC11646098; doi:10.1093/ckj/sfae349)
Supplement: sfae349_Supplemental_File [file sfae349_supplemental_file.pptx]

## Slide 1
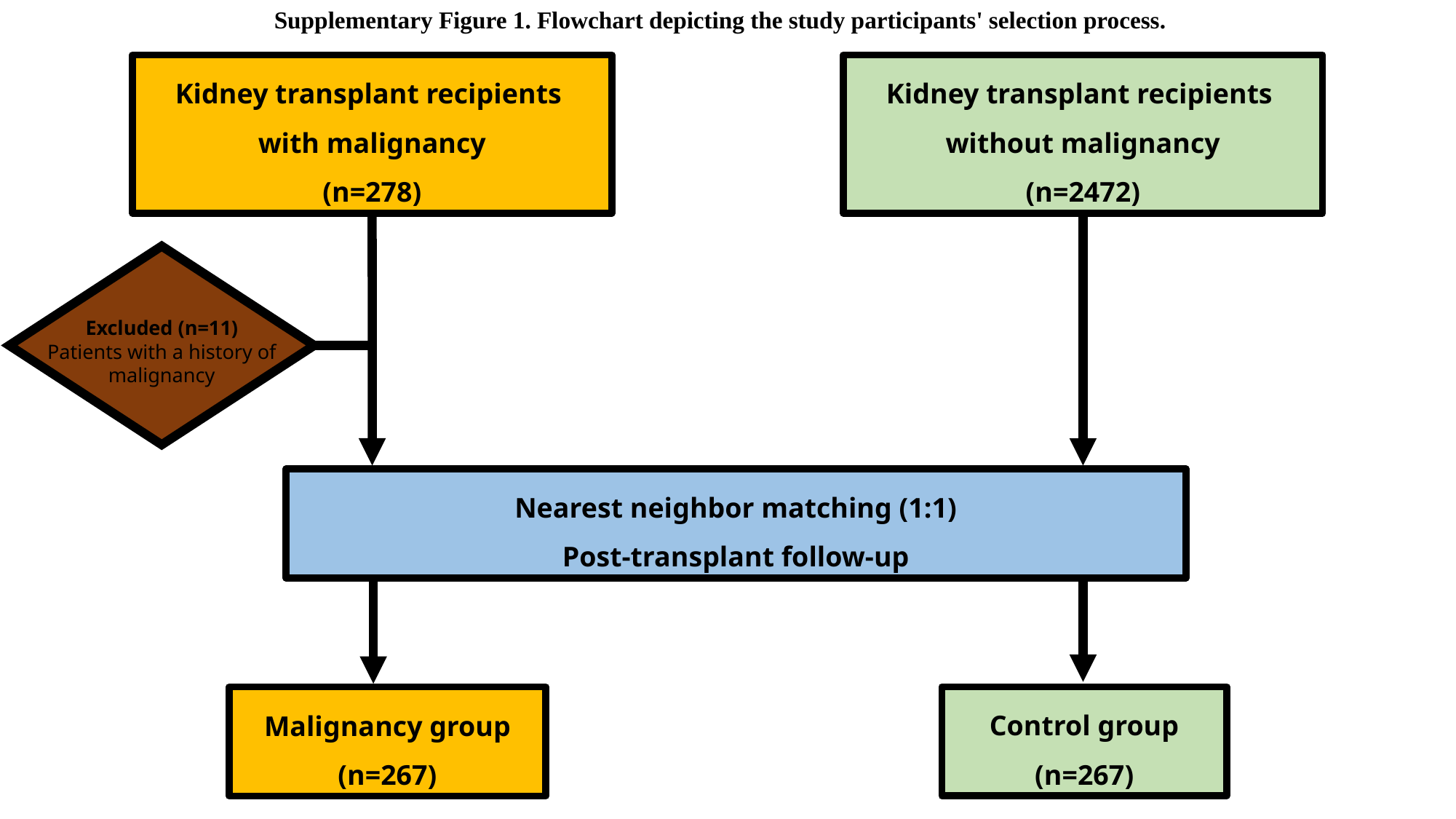

Supplementary Figure 1. Flowchart depicting the study participants' selection process.
Kidney transplant recipients
with malignancy
(n=278)
Kidney transplant recipients
without malignancy
(n=2472)
Excluded (n=11)
Patients with a history of malignancy
Nearest neighbor matching (1:1)
Post-transplant follow-up
Control group
(n=267)
Malignancy group
(n=267)

## Slide 2
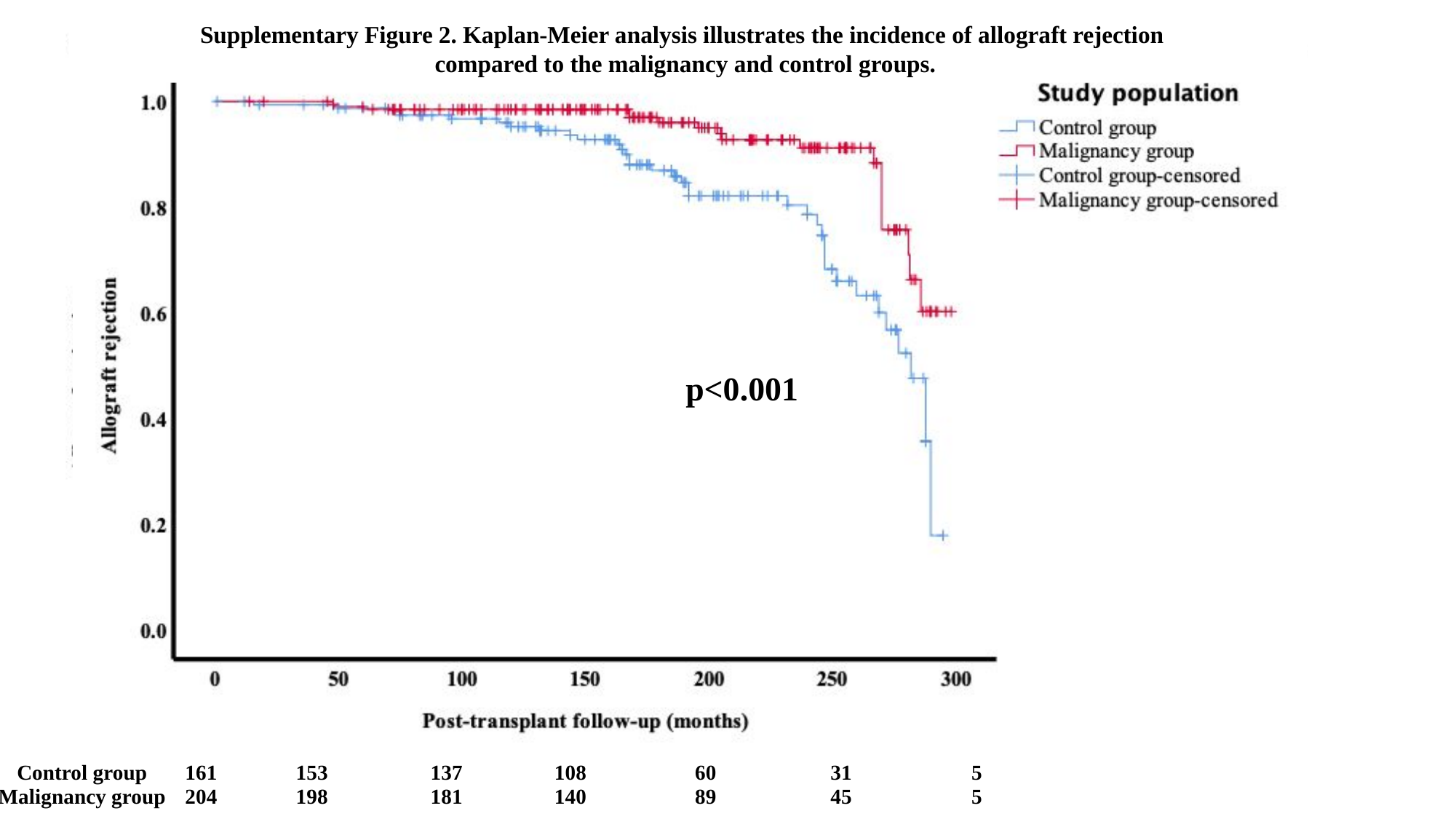

Supplementary Figure 2. Kaplan-Meier analysis illustrates the incidence of allograft rejection
compared to the malignancy and control groups.
p=0.34
p<0.001
| Control group | 161 | 153 | 137 | 108 | 60 | 31 | 5 |
| --- | --- | --- | --- | --- | --- | --- | --- |
| Malignancy group | 204 | 198 | 181 | 140 | 89 | 45 | 5 |

## Slide 3
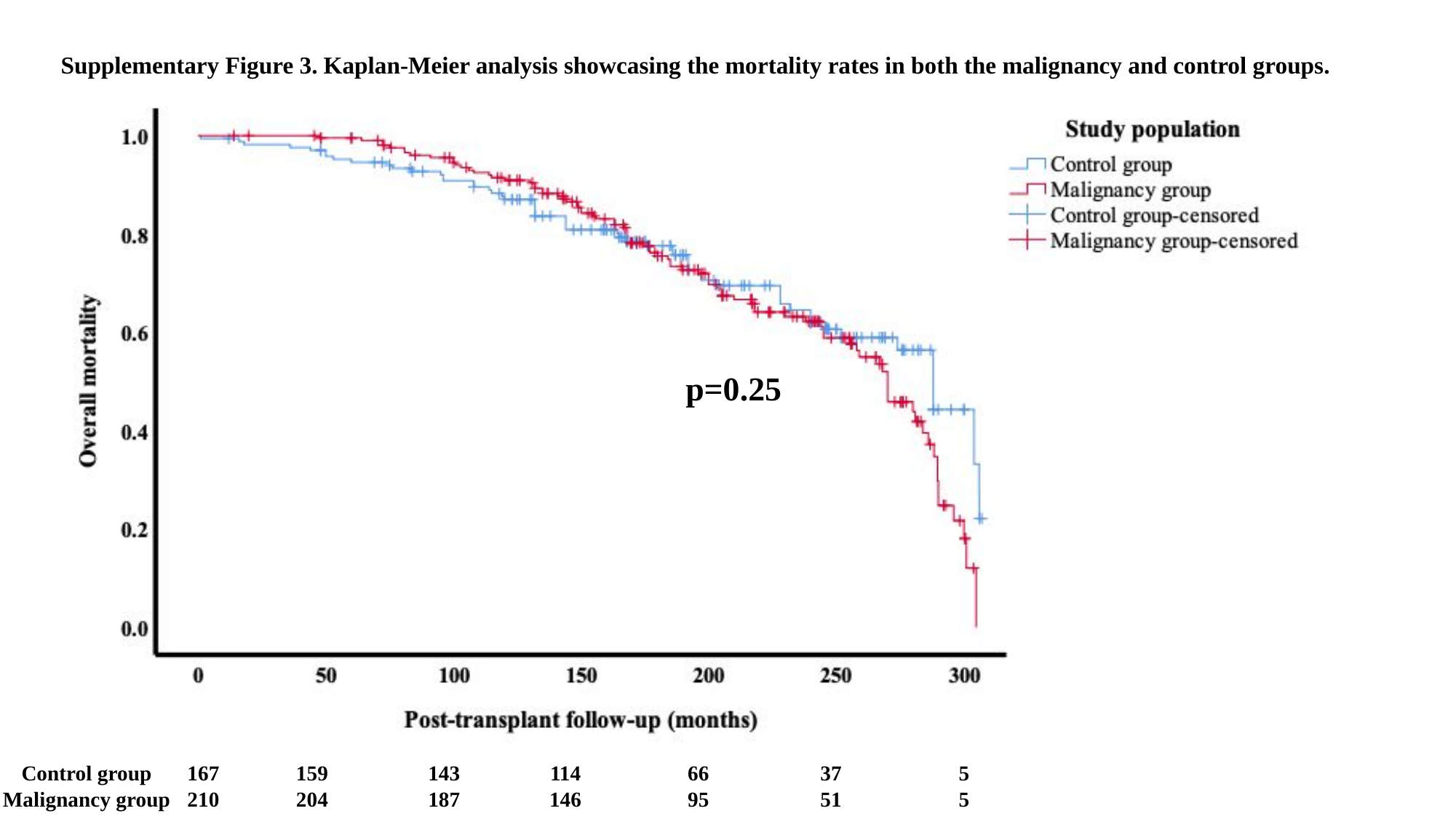

Supplementary Figure 3. Kaplan-Meier analysis showcasing the mortality rates in both the malignancy and control groups.
p=0.25
| Control group | 167 | 159 | 143 | 114 | 66 | 37 | 5 |
| --- | --- | --- | --- | --- | --- | --- | --- |
| Malignancy group | 210 | 204 | 187 | 146 | 95 | 51 | 5 |

## Slide 4
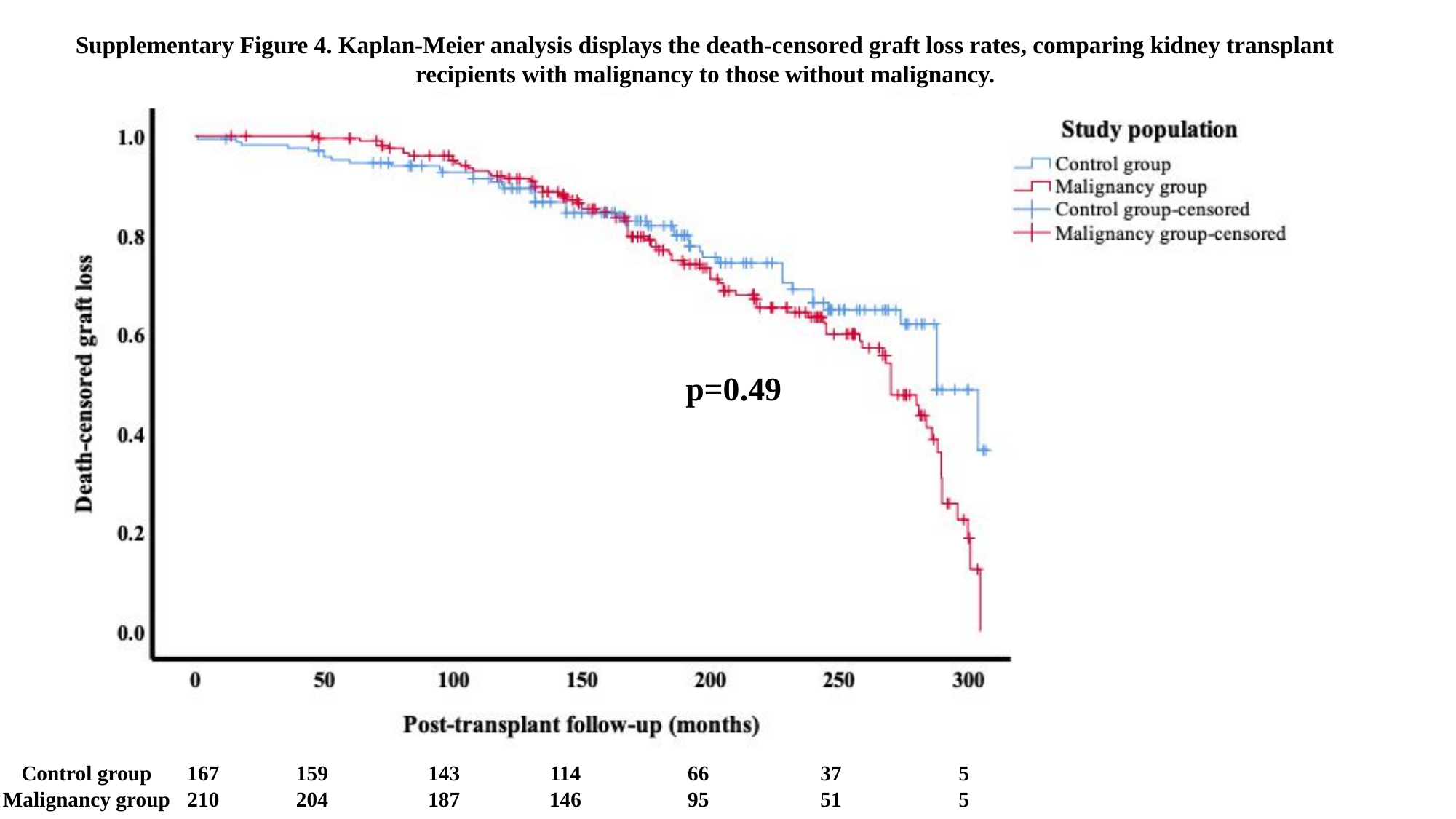

Supplementary Figure 4. Kaplan-Meier analysis displays the death-censored graft loss rates, comparing kidney transplant recipients with malignancy to those without malignancy.
p=0.49
| Control group | 167 | 159 | 143 | 114 | 66 | 37 | 5 |
| --- | --- | --- | --- | --- | --- | --- | --- |
| Malignancy group | 210 | 204 | 187 | 146 | 95 | 51 | 5 |
